# Metadata behind the Interoperability of Wireless Sensor Networks

**DOI:** 10.3390/s90503635

**Published:** 2009-05-14

**Authors:** Daniela Ballari, Monica Wachowicz, Miguel Angel Manso Callejo

**Affiliations:** 1 ETSI Topografía, Geodesia y Cartografía. Technical University of Madrid, Campus Sur UPM, Autovía de Valencia Km 7,5, E-28031 Madrid, Spain; E-Mail: m.manso@upm.es (M.A.M.C.); 2 Wageningen UR, Centre for Geo Information, Droevendaalsesteeg 3, 6708 PB, Wageningen, The Netherlands; E-mail: monica.wachowicz@wur.nl (M.W.)

**Keywords:** metadata, context model, contextualising rules, bridge rules, dynamic interoperability, wireless sensor network

## Abstract

Wireless Sensor Networks (WSNs) produce changes of status that are frequent, dynamic and unpredictable, and cannot be represented using a linear cause-effect approach. Consequently, a new approach is needed to handle these changes in order to support dynamic interoperability. Our approach is to introduce the notion of *context* as an explicit representation of changes of a WSN status inferred from metadata elements, which in turn, leads towards a decision-making process about how to maintain dynamic interoperability. This paper describes the developed context model to represent and reason over different WSN status based on four types of contexts, which have been identified as sensing, node, network and organisational contexts. The reasoning has been addressed by developing contextualising and bridges rules. As a result, we were able to demonstrate how contextualising rules have been used to reason on changes of WSN status as a first step towards maintaining dynamic interoperability.

## 1. Introduction

Sensors and their networks are becoming essential sources of information for planning, risk management and other scientific applications. They are revolutionising the way geo-referenced data is collected and analysed [1]. In this paper, the focus is on Wireless Sensor Networks (WSNs). These networks are composed of a large number of nodes, densely deployed within or very close to a phenomenon of interest [2]. They present an advantage over other sensor networks mainly because the WSN nodes are small, lightweight, and they consume less energy. They are usually self-adaptive systems and can be deployed with a spatial distribution that best fits the communication protocol requirements for gathering geo-referenced data [3]. Data collected by the nodes are typically transmitted through the wireless network to a sink node using some radio frequency technology, which supports the storage of the transmitted data and the communication with other devices and networks.

The interoperability of WSNs aims at the achievement of an integrated sensing system, in which sensors act in a collaborative and autonomous approach to produce more value than individual observations [4,5]. The objective of the sensor standardisation initiatives carried out by the Institute of Electrical and Electronics Engineers (IEEE) and the Open Geospatial Consortium (OGC) is to overcome the heterogeneity of devices, communication protocols, networks, data formats and structures. However, in order to support the interoperability of WSNs over time it is necessary to deal with the dynamic changes in the network, components and functionalities [6,7]. In general, interoperability could be achieved by taking into account several levels, including technical, syntactic, semantic, pragmatic, and dynamic ones [8]. For example: (a) the technical level of interoperability aims at the interconnection of WSNs using common communication protocols, hardware and software; (b) the syntactic level supports the exchange of information among WSNs using a common data structure, language, logic, records and files; (c) the semantic level supports the exchange of information using a common vocabulary and it is related to standards and specifications that define schemas for the exchange of information and meaning. In the case of the pragmatic interoperability level, it allows the interconnected WSNs to be known to each other and can explore interface applications and/or services to invoke methods or procedures in order to manage the data they need. Finally, the dynamic interoperability level allows the monitoring of operation of other WSNs and the response to changes.

Currently the OGC Sensor Web Enablement specifications (e. g. SML, SOS, SAS, SPS) provide a set of standards, interfaces and encodings to achieve interoperability of sensor and sensor systems [5]. From our understanding, it is mainly designed to handle the following interoperability levels: technical (web technologies), syntactic (encodings) and pragmatic (standardised interfaces). Moreover, some initiatives are being carried out to deal with the semantic interoperability of sensors [9,10]. However, the dynamic interoperability still remains to be addressed in order to monitor and manage the changes of the status of different WSNs over time. Some of these changes are due to internal factors, such as a battery run down or a neighbour's communication failure. Others may be produced by external factors such as node damage by weather conditions or changes of objectives, purpose, security and privacy constraints.

Therefore, the main research challenge is mostly related to the heterogeneity and dynamic issues of how to maintain the interoperability of WSNs over time. When a change of a WSN's status occurs, the system must respond by triggering a self-adaptive process, which in turn, is interconnected with the WSN's functionalities. These functionalities are used to configure, protect, optimise and repair a network itself, without the intervention of humans. They monitor the changes, detect failure or degradation of performance, begin diagnostic procedures, and conduct preventive, corrective and proactive actions [11]. However, in the case of maintaining dynamic interoperability, the monitoring of these functionalities is not a simple task. Mainly because the dynamic and unpredictable changes of a WSN's status cannot be represented using a linear cause-effect approach. For instance, usually if a node has a low level of energy, the action could be to “sleep” this node. But if this node is interoperating in an emergency situation (e.g. natural disaster, terrorist attack), then it must continue sensing and transmitting data instead of sleeping. This reasoning process of monitoring and adaptation needs to be *contextualised* because it depends on the context inside which the sensing is carried out [12].

Therefore, our research premise is the existence of different contexts, both at in-network and data repository levels, which play an important role in the dynamic interoperability of WSNs. From a pragmatic point of view, the dynamic interoperability of WSNs at different periods of time can be maintained by using a set of metadata elements in order to provide a description of observations, processes and functionalities, as well as their current configuration that can enable the understanding of a network itself [13-15]. Metadata are the common thread that can connect all the status and functionalities of WSNs as well as preserve the context of the sensing data [16,13]. This paper describes the development of a context model based on metadata elements for maintaining the dynamic interoperability of WSNs. The reasoning process to contextualise the dynamic interoperability of WSNs using metadata elements is carried out by two types of reasoning rules. One of them, the contextualising rule, is introduced in the scope of our research to identify different WSN status according to a specific context using metadata elements. The other type, called bridge rules, was previously introduced by Giunchiglia [12], and it is used to represent the relationship between contexts and the dynamic interoperability. It is important to point out that previous developed context models have mainly considered sensors as a mechanism to capture information about the context itself [17]. In contrast, this paper proposes a model focused on a context decision-making representation about how to maintain the sensor dynamic interoperability instead of only handling the WSN status changes.

The next section describes the concept of metadata and their principal requirements in the scope of WSNs. Section 3 describes what notion of context has been envisaged and why contexts are needed to reason about the WSN status changes in order to maintain the dynamic interoperability. The developed context model and the relevant aspects of its representation are discussed in Section 4. Furthermore, Section 5 describes the reasoning mechanisms of inferring and connecting contexts by providing examples of contextualising rules. Section 6 provides a discussion about the impact of the context model in WSN interoperability by providing examples of bridge rules. Finally, the main conclusions are summarised in Section 7.

## 2. The Notion of Metadata

The most widely used definition of metadata is “data about data”. They provide the description of the what, where, when, who and how about data [18]. A comprehensive metadata example is that of a photograph, in which the metadata describe when and where the photograph was taken, who the photographer was, what is in the photograph, what the camera features are or what post-processes have been done. The metadata are generally used to describe and structure the principal aspect of data with the aim of sharing, reusing and understanding heterogeneous data sets and allowing the information searching and retrieval [18,19]. In the scope of WSN, metadata have been defined as descriptive data used to depict the WSN, including the environment, the nodes and their status, sensing data, and the WSN as a whole system [16]. The use of metadata in WSNs has been mainly related with the execution of routing protocols and in-network data aggregation processes [20-22].

Currently, the metadata need to be become an *explicit* part of WSN in order to preserve the knowledge of the WSN's status over time (Table 1). On the one hand, they must describe dynamically the changes of the network status and report them back to other components and systems. For example, if a node changes its location or is damaged, the system must be able to broadcast a message containing metadata elements in order to inform other sensor networks and users about these changes. On the other hand, metadata must be automatically generated and updated, since real-time sensor data require real-time metadata as well. For example, if a node fails, the network must automatically (i.e. without human intervention) reconfigure new routes to send data. In the same way if a node changes its location, the sensing data (and their metadata) must reflect the new location.

## 3. The Notion of Context

**Figure 1.**
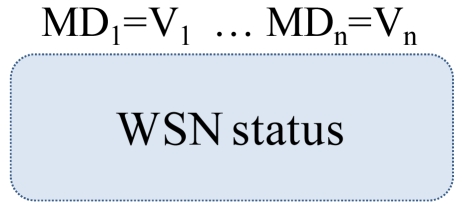
The box metaphor of a context.

Despite the large amount of research work in the field of Artificial Intelligence, there is no concise definition of a context [23]. This makes it difficult to select a logical structure of representation and reasoning when context-dependent information is involved, in particular the one generated by WSNs. In this paper, we have used the metaphor of a box as proposed by Giunchiglia and Bouquet [24]. In this case, a context is a box that can be divided into two parts (Figure 1):

- inside the box: a collection of WSN status that describes the status of a WSN over time,
- outside the box: a collection of metadata elements (MD) and their respective values (V).

The assumption is that the content of what is inside the box is determined by the values of the metadata elements associated with that box. In other words, the contexts of a WSN's status are inferred using metadata elements that describe the sensing system, the current network configuration, and the environment restrictions. To address the box metaphor into the dynamic interoperability of WSNs, two considerations must be made [25]. First, the dynamic WSN status (and its required self-adaptation) is considered as a local model, in the sense that the WSN's status is based on local information. This has to do with the relationship between metadata elements and their values, and the representation of a context inside the box. For example: How the metadata elements and their values affect the representation of a WSN's status? In what sense a metadata element provides implicit information which can be used to infer a context for interpreting what is inside the box? Second, the dynamic interoperability is considered as a global model in the sense that happens across multiples and heterogeneous WSNs and with multiple and shareable context representations. The issue here has to do with the relationship among the boxes. For example: How do these relationships affect the contents of different boxes? Therefore, the connection between global and local models can only be achieved by the representation and reasoning on different contexts. Contextualising WSN interoperability can be achieved by using reasoning rules, between dynamic interoperability (global model) and the WSN's status (local model).

**Figure 2.**
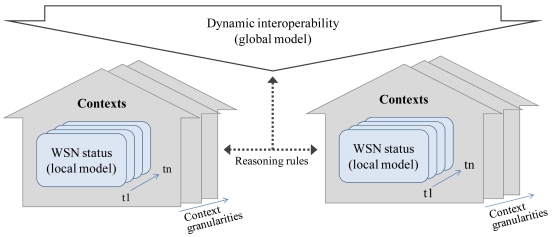
Contexts connecting WSN status with dynamic interoperability through reasoning rules.

The contexts are local (where local is intended here to imply not shared) models that encode a party's view of a domain [25]. In the scope of our research, the parties are the WSN that interoperate; the domain is the dynamic interoperability and the view of a domain is the current status that has influence over its dynamic interoperability. Thus, in our model *contexts are local models that represent the current WSN status in the domain of dynamic interoperability* (Figure 2).

Bouquet [25] also points out that the notion of context is best used in those applications where the core problem is the use and management of local and autonomous representations. This is particular the case of WSN applications. Moreover, contexts are easier to define and maintain. They can be constructed with no consensus among different WSNs, or only with the limited consensus which make it possible to achieve the desired level of communication. On the weak side, since contexts are local to WSNs, communication can be achieved only by constructing explicit mappings among the metadata elements of the WSNs' contexts. In a contextualised WSN, the knowledge is kept locally, but it could be put in relation with the knowledge of other WSN's contexts and the global model via explicit mapping. Moreover, the context of a WSN is not unique in the sense that multiples contexts could be inferred for the same WSN's status. It could be described with different granularities based on the different levels of approximation, perspectives or temporal considerations.

Finally, we distinguish two types of reasoning rules that are involved in the contextualisation of WSN interoperability: (a) *Contextualising rules*, they are used to infer the contexts of WSN status when WSN metadata and their values are matched by the rules. Following the box metaphor, they allow the interpretation of what is happening inside the box; and (b) *Bridge rules*, that allow the relationships among different boxes in order to connect different WSN status with the dynamic interoperability. They can modify what happens inside a box depending on the inferred contexts in other box. However, in this paper we focus on the first of them, contextualising rules, and they are showed in more detail in Section 5.

## 4. The Developed Context Model

The context model represents the knowledge of the current WSN status, through the status of the sensing functionality, the node, the network and the organisational features. Inspired by the compone-and-conquer approach [26], we have defined our context model based on four types of contexts. They are: sensing, node, network, and organisational contexts. Furthermore, there are relationships among these types of contexts representations that enable the implementation of contextual reasoning to compose a more understanding and compressive view of dynamic interoperability. Table 2 includes some examples of the four types of contexts.

### 4.1. Sensing Contexts

These are the representations used to generate the knowledge about the context in which the data are being captured. They describe the sensing conditions, the sensing operations, and help to evaluate and understand the sensing data [27]. In order to infer the knowledge about these contexts some metadata elements are needed. The metadata elements would contain (a) the spatial information, such as the sensor and data location and spatial reference system; (b) the temporal information, such as instant time or interval of observation; and (c) thematic information, such as feature of interest and phenomena [9]. Other descriptive metadata elements are related to the data capture and observation processes, data collection characteristics (periodic, continuing, or reactive). The inferred knowledge could be related with “when” the data are sensing (day, night, season), “where” (sea, mountain, forest), “how” (sensing process, sensors) and “what” (phenomena, feature of interest). For instance, a WSN node is attached to a bike and it must monitor only when the bike is moving. When it infers from the variation of GPS or accelerometer data that the bike is moving, the monitoring system is started. In our model, this WSN status in which the movement could be inferred from sensing data is represented by the *mobile phenomena context*.

### 4.2. Node Contexts

These are the representations used to generate the knowledge of individual nodes that compose the network. In a field deployment, the interoperability will happen at node level. The nodes could be able to participate in collaborative tasking through different networks, such as data transmission processes, and in-network data aggregation. The metadata elements related with this context describe the state of memory, communication devices, sensors, actuators, and processor for each individual node. The inferred knowledge will be in concordance with the node status at a specific time and its impact on the interoperability with other nodes. For example, in a mobile WSN in which nodes are moving freely, communication failure is common when nodes do not have near neighbours. Later, when the node recovers its neighbour's nodes, the communication will be recovered too. The interoperability will be interrupted while the node is without neighbours. The node must know his context and must act based on this context. In our model this particular node status is represented by the *isolation context*.

### 4.3. Network Contexts

These are the representations used to generate the knowledge about the functionalities, collaboration and interrelations among nodes. They represent node collaborations in communication and processing functionalities to configure the wireless sensor network. They are related with the current configurations of interoperable networks. The metadata elements used in this context are dynamic and some of them could derive from the node contexts as emergent properties of the network. Some contexts examples are the network composition (homogeneous, heterogeneous), organisation (hierarchical, flat), density (balanced, densely spaced), distribution (regular, irregular), size (small, medium, large), residual network energy and memory (low, high), and sensing coverage area (insufficient, exceeded). In the nodes mobility example, the predetermined coverage area could be exceeded or insufficient covered by the nodes. This context must be known in order to trigger adaptive processes to cover in an efficient way the assigned area. In our model these network status are represented by the *exceeded coverage area context* and the *insufficient coverage area context*.

### 4.4. Organisational Contexts

These are the representations used to generate the knowledge about objectives, and legal, security and privacy restrictions. They show the policies behind the WSN's performance and how it can interoperate with other WSNs or devices. For instance, the interoperability of a WSN may be forbidden for security reasons; or certain nodes can have limitations to interoperate because of restrictions imposed to conserve their energy. If the WSN accesses an area with a different security code, then the WSN must act restricting its interoperability in concordance with the new security level. In our model these organisational status are represented by the *high, medium and low security level contexts*.

### 4.5. Relevant Aspects of the Context Model

After analysing these proposed contexts, we are able to include some relevant aspects about how the context model is represented. They are described as one of the following:

#### 4.5.1. The contexts have different dynamics

The dynamic of the changes in the four types of contexts is not the same. The node and network contexts present more dynamic status changes and these are unpredictable. Meanwhile, the sensing and organisational context are more static, in the sense of their changes are less usual, and they are mainly carry out by a human intervention. The sensing, node and network contexts are associated with the network itself, while the organisational context is associated with non-physical aspects of the WSN.

#### 4.5.2. The contexts depend on the metadata values

The metadata elements characterise the WSN's status, or in other words, the WSN's status is obtained using metadata. But these metadata are not previously assigned to the contexts. Thus, the contexts could change depending on the metadata values. An example is related with the NodeNeighbours metadata element (Table 3). If the NodeNeighbours = 12, then the network context will be high density of nodes. In this context the node will select the best path to send the sensing data to the sink node. Meanwhile, if the NodeNeighbours = 2, the network context will be low density of nodes. Finally if the NodeNeighbours = 0, the context will be isolation node and the interoperability will be interrupted. The node must adapt itself in order to overcome this status and to avoid the sensing data lost.

#### 4.5.3. The contexts depend on the level of approximation

As an example we will use an essential context component: the location or “where”. The meaning of “where” could change according to the level of approximation (Table 4). If a node has a GPS sensor, it will be possible to attach the spatial coordinates to other sensor measures such as temperature, light, humidity. This spatial location will belong to the sensing contexts. On the other hand, if the GPS sensor is tracking the node trajectory, the observed location becomes a part of the node contexts. Based on the individual node location it will be possible to define the network coverage area, the extension, density, parent location, encounters between nodes, and detention areas that belong to a network context.

#### 4.5.4. The contexts have relationships among them

These relationships are based on bridge rules. They link different contexts when the knowledge inferred in one context has influence in the knowledge of another context [12]. The bridge rules allow the mapping of multiple WSN's contexts [26]. For instance, to compute the network coverage area (network context) is necessary to know the position of the nodes (node context). On the other hand, for security reasons only authorised systems (organisational context) are allowed to access to certain sensing functionalities (sensing context). The fixed/mobile contexts (node context) could be inferred from the GPS or accelerometer data (sensing context). Furthermore, any node interaction must be validated by the security and privacy restriction (organisational context).

#### 4.5.5. Run-time and historic time contexts

From the temporal consideration, we could distinguish between two kinds of context, the run-time and the historic time. The run-time context is the context corresponding with the current WSN status, and it is used in real-time. Meanwhile, the historic time context is the “memory” of previous status. In the isolation case context, when this context is inferred the system will trigger in-node storage process to avoid the sensing data lost while the node is in isolation context. When the neighbour communication is restored a new context will be inferred: *in-network node*. But the node must have “memory” in order to know what data was stored in-node during the previous context (isolation context) and must be sent to the sink node with the multi-hop protocol. Additionally, the sensing contexts use the historic time context to preserve the contents of the sensing data.

## 5. Reasoning about WSN Contexts: the Implementation of Contextualising Rules

Different forms of contextual reasoning are involved to carry out the reasoning mechanisms of inferring and connecting contexts. Benerecetti, in his work about the foundation of a theory of contextual reasoning, identifies three fundamental dimensions of contexts (*partiality*, *approximation* and *perspective*) and their relation with three forms of contextual reasoning (*localised*, *push and pop* and *shifting reasoning*). Thus, depending on the context dimension different mechanisms of context reasoning will be used [23].

If the focus is on the *partiality* context dimension the reasoning mechanism will be *localised* reasoning. The partiality is the portion of domain that is represented, and in our case it is composed by the four types of contexts: sensing, node, network and organisational contexts. The localised reasoning does not consider all that is known about a domain, but rather a subset of the knowledge [12,23]. In this approach, the reasoning is kept locally based on the local WSN status, and it is linked with other WSN's status and with the dynamic interoperability (global model) using the bridge rules. For instance, if the local context of a node is low level of energy, the consequence could be to sleep the node. But if this context is connected with an emergency context, then the node must continue sensing instead of sleeping. The inference process in the dynamic interoperability domain could generate different knowledge and different decision-making actions depending on the local and global models.

Moreover, when the contexts depend on the level of approximation it is possible to change the contexts granularity by adding (pushing) or extracting (popping) some metadata elements into the context box. For instance the “where” context could change according if the approximation is at sensing, node, network, or organisational contexts. Then, adding node location contexts (individual node locations) will determine the network location context (network coverage area). Thus, if the focus is on the *degree of approximation*, the reasoning about WSN contexts will be *push and pop* reasoning.

Finally, if the focus is on the changing the value of metadata (*perspective* dimension) the reasoning about WSN contexts will be a *shifting* reasoning. This form of reasoning is called shifting because the changing of metadata value shifts the WSN contexts. For instance, when the NodeNeighbours metadata change its value from 12 neighbours to 0 neighbour, the perspective from which the WSN is observed changes radically from a high density context to an isolation context.

In our approach the WSN contexts are inferred from the WSN's status using metadata elements. Therefore, we introduce contextualising rules to reason over WSN status using data and metadata that describe the sensing system, the current network configuration, and the environment restrictions. The contextualising rules are deductive rules (*if-then-else)* and are fed by the current WSN metadata. Some of these metadata are static and established by default (e.g. access restrictions, security level, and owner). Meanwhile, others are dynamic and automatically extracted from the WSN (e.g. energy level, node location). The dynamics of a WSN's status should be automatically captured and self-described through metadata and some of them can be derived by the data itself (e.g. the accelerometer data help to infer if the node is moving or fixing).

**Figure 3.**
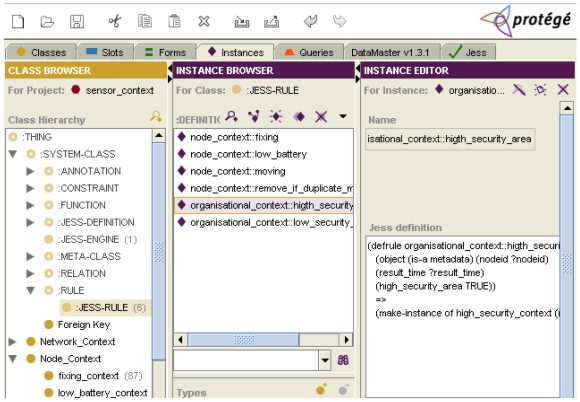
Contextualising rules implemented with Jess in Protégé.

The implementation of the contextualising rules has been achieved by using the Jess rule engine (Figure 3). Jess is a rule-based system that uses rules to derive conclusions from premises. The premises are the *if* first part of rules, meanwhile the conclusions are the *then* second part of rules. The Jess architecture consists of (a) the rule base that contains all the defined rules, (b) the working memory that is the WSN metadata elements and their values (also called facts) that the rule engine will operate on, and (c) the inference engine that controls the process of firing the rules and matching them with the working memory. We have used the Jess rule engine integrated into the Protégé knowledge-engineering framework, though the JessTab plug-in [28,29]. This has allowed us to develop the mapping between the Protégé knowledge bases (context classes) and Jess facts (metadata elements and their values). In our implementation, when a new set of metadata instances are uploaded in Protégé, the contextualising rules are executed and, as a result, the current contexts are inferred according to their current metadata values.

In the next section, we show some examples of contextualising rules expressed in Jess language to illustrate how they work. In these examples contexts are inferred and the interoperability must adapt to these new contexts in order to continue interoperating. They are simple rules constructions but they are useful to illustrate how contexts could be inferred from: the automated extracted metadata (Example 1), the sensing data (Example 2) and the metadata extracted using the GPS sensing data (Example 3).

### 5.1. Example 1

This rule uses the battery level metadata to infer if the node is sensing in a low battery context. It is a useful context in WSNs in which resource optimisation is adapted depending on, for example, if the node must sleep, or on the other hand, it must continue sensing because the context of interoperability is an emergency situation. The Jess rule engine evaluates the metadata load into its working memory, and when they are matched by the premise “if the battery level is less or equal than a defined threshold (<= ?battery threshold)”, then the node is classified into the low battery context.

(1) $$\begin{array}{c} (\text{defrule node}\_\text{context}∷\text{low}\_\text{battery}\\ \left(\text{object}(\text{is}-\text{a metadata})(\text{nodeid}?\text{nodeid})(\text{result}\_\text{time}?\text{result}\_\text{time}\right)\\ \left(\text{battery}?\text{battery}\&:(<=?\text{battery threshold}))\right)\\ =>(\text{make}-\text{instance of low}\_\text{battery}(\text{nodeid}?\text{nodeid}()\text{result}\_\text{time}?\text{result}\_\text{time}))) \end{array}$$

### 5.2. Example 2

In this example, two contextualising rules are developed to infer whether the context of a node is fixed or mobile. When the nodes are mobile, the WSN status changes will be more frequent and sometimes unpredictable. Thus, the system must increase its monitoring over communication, coverage area, and network topology in order to detect relevant changes of status for the purpose of interoperability. For example if the node has left the coverage area of interest, the interoperability will be interrupted. In this case, the fixed and mobile contexts are defined using the accelerometer sensing data. When the accelx and accely data match the defined threshold value, the rules classify the node into a fixing or moving contexts. Finally, an additional rule is fired to validate that there are not duplicated instances.

(2) $$\begin{array}{c} (\text{defrule node}\_\text{context}∷\text{fixing}\\ \left(\text{object}(\text{is}-\text{a sensing}\_\text{data})(\text{nodeid}?\text{nodeid})(\text{result}\_\text{time}?\text{result}\_\text{time}\right)\\ \left(\text{accely}?\text{accely}\&:(\text{and}(>=?\text{accely threshold})(<=?\text{accely threshold}))\right)\\ \left(\text{accelx}?\text{accelx}\&:(\text{and}(>=?\text{accelx threshold})(<=?\text{accelx threshold})))\right)\\ =>(\text{make}-\text{instance of fixed}\_\text{context}(\text{nodeid}?\text{nodeid})(\text{result}\_\text{time}?\text{result}\_\text{time})\\ \left(\text{accely}?\text{accely})(\text{accelx}?\text{accelx}))\right) \end{array}$$

(3) $$\begin{array}{c} (\text{defrule node}\_\text{context}∷\text{moving}\\ \left(\text{or}(\text{and}(\text{object}(\text{is}-\text{a sensing}\_\text{data})(\text{nodeid}?\text{nodeid})(\text{result}\_\text{time}?\text{result}\_\text{time}\right)\\ \left(\text{accely}?\text{accely}\&:(\text{or}(<?\text{accely threshold})(>?\text{accely threshold})))(\text{accelx}?\text{accelx}))\right)\\ \left(\text{and}(\text{object}(\text{is}-\text{a sensing}\_\text{data})(\text{nodeid}?\text{nodeid})(\text{result}\_\text{time}?\text{result}\_\text{time}\right)\\ \left(\text{accely}?\text{accely}\right)\\ \left(\text{accelx}?\text{accelx}\&:(\text{or}(<?\text{accelx threshold})(>?\text{accelx threshold})))))\right)\\ =>(\text{make}-\text{instance of moving}\_\text{context}(\text{nodeid}?\text{nodeid})(\text{result}\_\text{time}?\text{result}\_\text{time})\\ \left(\text{accely}?\text{accely})(\text{accelx}?\text{accelx}))\right) \end{array}$$

(4) $$\begin{array}{c} \left(\text{mapclass moving}\_\text{context}\right)\\ (\text{defrule remove}\_\text{if}\_\text{duplicate}\_\text{instances}\_\text{moving}\_\text{contex}\\ \left(\text{object}(\text{is}-\text{a moving}\_\text{context})(\text{nodeid}?\text{nodeid})(\text{result}\_\text{time}?\text{result}\_\text{time}\right)\\ \left(\text{object}?\text{instance})\right)\\ \left(\text{object}(\text{is}-\text{a moving}\_\text{context})(\text{nodeid}?\text{nodeid})(\text{result}\_\text{time}?\text{result}\_\text{time}\right)\\ \left(\text{object}∼?\text{instance})\right)\\ =>(\text{unmake}-\text{instance}?\text{instance})) \end{array}$$

### 5.3. Example 3

In order to infer if a node is sensing in a high or low security geographical area, we use a WSN node with a GPS sensor. When the nodes access an area with a different security level, they must act by restricting its interoperability in accordance with the new security level. For example certain phenomena will be forbidden to sense or to access in public areas due to privacy issues. In practice, the GPS sensing data was converted into a spatial database using the PostGIS spatial extension of the PostgreSQL object-relational database. PostGIS allows GIS (Geographic Information Systems) objects to be stored in the database and includes functions for the analysis and processing of spatial objects, such us proximity, adjacency or containment [30]. Thus, the metadata provide information about where the nodes are located, and whether the containment is in a high or low security area. When these spatial metadata have been extracted, the rule engine is fired and the high security and low security context are inferred.

(5) $$\begin{array}{c} (\text{defrule organisational}\_\text{context}∷\text{high}\_\text{security}\_\text{area}\\ \left(\text{object}(\text{is}-\text{a metadata})(\text{nodeid}?\text{nodeid})(\text{result}\_\text{time}?\text{result}\_\text{time}\right)\\ \left(\text{high}\_\text{security}\_\text{area TRUE})\right)\\ =>(\text{make}-\text{instance of high}\_\text{security}\_\text{context}(\text{nodeid}?\text{nodeid})(\text{result}\_\text{time}\\ ?\text{result}\_\text{time}))) \end{array}$$

(6) $$\begin{array}{c} (\text{defrule organisational}\_\text{context}∷\text{low}\_\text{security}\_\text{area}\\ \left(\text{object}(\text{is}-\text{a metadata})(\text{nodeid}?\text{nodeid})(\text{result}\_\text{time}?\text{result}\_\text{time}\right)\\ \left(\text{low}\_\text{security}\_\text{area TRUE})\right)\\ =>(\text{make}-\text{instance of low}\_\text{security}\_\text{context}(\text{nodeid}?\text{nodeid})(\text{result}\_\text{time}\\ ?\text{result}\_\text{time}))) \end{array}$$

## 6. The Impact of the Proposed Context Model in WSN Interoperability

In this paper we have mainly focused on the definition of local context model based on metadata elements. However, it is still necessary to address the development of a global model for achieving the dynamic interoperability of WSNs. Our proposed local model addresses issues such as mobility, entrance and exit of nodes in network, energy levels, network configuration and topology, privacy and security constrains. We argue that based on the WSN inferred contexts it will be possible to maintain the dynamic interoperability when unpredictable changes of status may occur. In other words, the WSNs will be monitored and when changes of status are detected, then current contexts and their responses will be inferred at the local model, and as a result, a decision making will be taken at the global model. This will be carried out based on the knowledge of WSN contexts over time that will allow to make a more intelligent decision based not only in the location and technical specifications of sensors, but also on the purpose of interoperability, security and privacy constraint, the environment in which the sensing and interoperability takes place and the current status of network.

**Figure 4.**
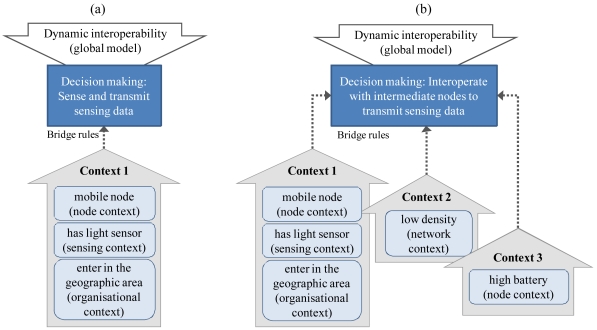
(a) Bridge rules evaluating local contexts. (b) Bridge rules linking and evaluating multiple contexts.

In fact, the contexts will provide the explicit knowledge about what happens in the WSN and in its surroundings; meanwhile, the bridge rules will be the reasoning mechanism that relates the contexts of different WSNs, and at the global model, a decision-making action will take place in order to decide what should be done to continue interoperating in despite of the dynamic changes. When the local contexts are inferred, they could be linked and evaluated using bridge rules. For example, the interoperability was established in a geographic area for solar luminosity monitoring. Then, when mobile nodes with a light sensor enter in this area, they will begin to sense and transmit this phenomenon (Figure 4a). But if the node density is low, the sensing data could not be transmitted in real time due the insufficient number of nodes. Thus, they will interoperate with other nodes using them as intermediate nodes to transmit the sensing data in real time, evaluating previously if their battery levels are high (Figure 4b).

The use of contexts in sensor interoperability tends towards an adaptive interoperability. For example when a WSN begins to interoperate with other sensors, its energy context could be high. Later it could be low and the interoperability is interrupted. On the other hand, if the interoperate purpose is an emergency situation (hurricane, flood, fire), the nodes will continue sensing. Other example with different interoperate purpose is a WSN transported by people and the nodes must interoperate exchanging some parameters if the people interact. Thus, when the proximity interaction context is inferred the nodes will interoperate. The criteria of these reasoning processes based on multiples local contexts and global interoperate purpose will be defined in our future research work.

## 7. Conclusions

In order to handle changes in WSN status and to support dynamic interoperability, the relationships between local and global interoperability models must be addressed. Towards this challenge we have introduced the notion of contexts as an explicit representation of WSNs' status inferred from metadata elements. Moreover, a context model is proposed to represent the WSNs' status based on four types of contexts: sensing, node, network and organisational. The focus in this approach has been on the capturing and reasoning over different contexts, using two types of reasoning rules: contextualising rules and bridge rules. However, in this paper we have focused on the development of contextualising rules as a mechanism to infer different WSN contexts from WSN status using metadata elements. As a proof of concept we have demonstrated examples of contextualising rules based on the localised reasoning in the node and organisational contexts, as well as on the shifting reasoning in which the contexts depend on the metadata value.

We have shown the important role of metadata elements to contextualise the dynamic interoperability of WSNs. The metadata act as parameters in order to interpret what is happening inside the box (i.e. the context). Depending on their value and the level of approximation, the interpretation of contexts could be different. Some people may argue that metadata are low level information about WSNs, but managing them in a properly form (contextualising rules), they allow the inference of high level of knowledge about the possible WSN contexts in which the sensing is carried out. The use of spatial metadata, such as location, coverage area or security area, adds the spatial dimension into the reasoning process allowing the inference of spatially related contexts.

Furthermore from sensing data collection view point, sensor networks are sensing a massive amount of data and with their interoperability the amount of data increase even more. Currently, all these data is provided in isolation without context [5]. Thus, contextualising the interoperability will allow a more intelligent recovery of sensing data and resources based not only in queries of where (geographic coordinates), when (date and time), how (sensor specification) or what (phenomena type), but also related with more rich contextual knowledge such us: all the nodes that are sensing in a high security areas or all the nodes that are sensing near the sea; the nodes that are sensing in the same context but not necessary in the same geographic context, the context in which two nodes had been interacted, all the nodes that are allowed to interoperate and are attached to public transport. The context-based information retrieval could be pointed out as an important issue of Sensor Web.

This paper describes our first step towards the maintenance of WSN interoperability. In order to contextualise the WSN interoperability a further analysis on the relationships among contexts is needed in order to develop the representation and bridge rules of the global model (dynamic interoperability). Therefore, our next research will be to implement the bridge rules as part of a decision-making process that can allow the reasoning among different contexts and between contexts and the dynamic interoperability, which in turn will allow us to decide what should be done to maintain the dynamic interoperability in despite of the changes of WSN status. We are planning to explore more in detail the localised, push and pop and shifting reasoning tasks and their relation with the bridge rules. Finally, we will implement a concrete case of study for the evaluation of our context model as an approach to address the dynamic interoperability of WSNs.

## Figures and Tables

**Table 1.** Examples of WSN metadata elements for temperature data.

**Data**	**Metadata Elements (MD)**	**Value (V)**
T = 22	Phenomena	Temperature
	Data unit	Celsius degree
	Time Result	2009/01/23 19:23:45
	Location	Lat 40°26′North; Long 3°42′West
	Feature of Interest	Technical University of Madrid Campus
	Mote type	mts420 crossbow
	Sensor Type	Sensirion SHT11
	Other data associated	Humidity, Barometric Pressure, Ambient, Light Sensors, Dual-Axis Accelerometer, GPS position.
	Node identifier	5
	Number of nodes in network	11
	Number of node neighbours	7
	….	….

**Table 2.** The four types of contexts in WSN interoperability.

**Sensing Contexts**	**Node Contexts**	**Network Contexts**	**Organisational Contexts**
same/different phenomena mobile phenomena indoor/outdoor	lack of resources fixed/mobile node isolation sleep/wake up	low/high density big/small network exceeded/insufficient coverage area	high/medium/low security restrictions avoid interoperability where (administrative area)

**Table 3.** Example of contexts depending on metadata values.

**Metadata Element**	**Values**	**Contexts**
NodeNeighbours	12	high density
	2	low density
	0	isolation

**Table 4.** Example of contexts according to different levels of approximation.

**Metadata Element**	**Level of Approximation**	**Contexts**
Location	Data location	Sensing Context
Lat 40°26′North	Node location	Node Context
Long 3°42′West	Network coverage area	Network Context
	Administrative area	Organisational Context
